# Dysmenorrhœa

**Published:** 1842-07-16

**Authors:** 


*Dysmenorrhœa.*—As a general rule, in women of a plethoric habit of body, repeated venesections will occasionally bring on the menstrual secretion; nervous women require sedatives and anti-spasmodics; the weak and chlorotic stimulants and tonics, especially chalybeates. When dysmenorrhœa is caused by any disease, that disease must be removed before the menstrual secretion can be reproduced.—*Ibid., from Examinateur Medical.*

